# 

**Published:** 1892-10-22

**Authors:** 


					HOSPITAL. ' r T) October 22nd, ]
. PRlOC TU/AElCKinC WW WITH EXTRA NURSING SUPPLEMENT
Ho Si 719/E. vT,W?PAt?0AO? M 1?^ Per Annum. 8s. 6d.t post free.
ra ? ') Vol. XIII. Oct. 22nd, 1892. I Monthly Parts, 6d.; post free, 8d,
SSS^SJ^SIfe^SHK''" ^ Ditto per Annum, 8s., post free>
No. 317. Vol. XIII.] Saturday,
October 22nd, 1892.
("Twopence.
n

				

